# Ingestion of a moderate dose of alcohol enhances physical exercise-induced changes in blood lactate concentration

**DOI:** 10.1590/1414-431X20209200

**Published:** 2020-04-06

**Authors:** F. Teixeira-Coelho, D.F.C. Santos, G.A. Santos, T.F. Sousa, S.R. Moreira, M.V.C. Souza, S.P. Wanner

**Affiliations:** 1 Centro de Formação de Professores, Universidade Federal do Recôncavo da Bahia, Amargosa, BA, Brasil; 2 Departamento de Ciências do Esporte, Instituto de Ciências da Saúde, Universidade Federal do Triângulo Mineiro, Uberaba, MG, Brasil; 3 Programa de Pós-Graduação em Educação Física, Universidade Federal do Vale do São Francisco, Petrolina, PE, Brasil; 4 Laboratório de Fisiologia do Exercício, Escola de Educação Física, Fisioterapia e Terapia Ocupacional, Universidade Federal de Minas Gerais, Belo Horizonte, MG, Brasil

**Keywords:** Endurance, Ethanol, Glucose, Lactate, Metabolism, Treadmill running

## Abstract

The consumption of alcoholic beverages influences carbohydrate and lipid metabolism, although it is not yet clear whether metabolism during physical exercise at different intensities is also affected. This was the objective of the present study. Eight young and healthy volunteers performed a treadmill test to identify the running speed corresponding to a lactate concentration of 4 mM (S4mM). At least 48 h later, they were subjected to two experimental trials (non-alcohol or alcohol) in which they performed two 1-km running sessions at the following intensities: 1) S4mM; 2) 15% above S4mM. In both trials, blood lactate, triglycerides, and glucose concentrations were measured before and after exercise. The acute alcohol intake increased triglycerides, but not lactate concentration under resting conditions. Interestingly, alcohol intake enhanced the exercise-induced increase in lactate concentration at the two intensities: S4mM (non-alcohol: 4.2±0.3 mM *vs* alcohol: 4.8±0.9 mM; P=0.003) and 15% above S4mM trial (P=0.004). When volunteers ingested alcohol, triglycerides concentration remained increased after treadmill running (e.g., at S4mM - at rest; non-alcohol: 0.2±0.5 mM *vs* alcohol: 1.3±1.3 mM; P=0.048). In contrast, glucose concentration was not modified by either alcohol intake, exercise, or their combination. We concluded that an acute alcohol intake changed lactate and lipid metabolism without affecting blood glucose concentration. In addition, the increase in lactate concentration caused by alcohol was specifically observed when individuals exercised, whereas augmented triglycerides concentration was already observed before exercise and was sustained thereafter.

## Introduction

For several years, the consumption of alcohol before any physical exercise was believed to have an ergogenic effect (1). However, since the publication of the official Position Stand of the American College of Sports Medicine (ACSM), prior alcohol consumption was associated with a reduction in endurance, among other physical capacities (2). Despite this, studies investigating the effects of pre-exercise alcohol intake on aerobic performance presented contradictory results: some of these studies reported no changes (3,4), whereas others have reported reduced performance (5–7). Probably, these contradictions can be explained by the different doses of alcohol administered, exercise protocols used, and alcohol tolerance among individuals (8).

While McNaughton and Preece (5) did not present an underlying mechanism to explain the ergolytic effect that was observed, Kendrick et al. (6) attributed the impaired aerobic performance to the hypoglycemic effect of alcohol. Other possible factors that may explain the decrease in performance are the metabolic effects of alcohol in the Cori cycle (9), citric acid cycle, pyruvate-to-lactate ratio, and availability of carbohydrates (10). Thus, it is likely that ingesting a moderate dose of alcohol prior to exercise may interfere with the exercise-induced changes in glucose uptake and in lactate production and removal pathways. In this sense, the focus of the present study was to investigate possible changes in metabolic parameters during exercise after the ingestion of an alcohol beverage.

A study carried out by Lecoultre and Schutz (7) was the only one that investigated simultaneously the effect of prior alcohol consumption on blood lactate and glucose concentrations during aerobic exercise, but observed no clear effect of alcohol on the concentrations of these metabolites/substrates that could explain the reduction in performance. As lactate concentration was similar between the two experimental trials (i.e., after intake of an alcoholic or a non-alcoholic control drink), in spite of the reduced power output during the time trial after alcohol consumption (7), we believe that if the power output was equal in both trials, the lactate concentration would be higher following alcohol consumption. Therefore, to reveal the effect of alcohol consumption on blood lactate concentration during exercise, it would be more appropriate to adopt an exercise protocol with fixed and similar relative intensity in the two trials. By adopting this strategy, we hypothesized that lactate concentration would be higher in the alcohol intake trial relative to the trial with no alcohol intake.

Multiple recent population studies confirm that chronic alcohol consumption is associated with changes in lipid metabolism (11,12), as evidenced by a linear relationship between alcohol consumption and plasma triglycerides concentration. Interestingly, a J-shaped association has also been described (13,14), with a decrease in plasma triglycerides being reported after chronic low-dose alcohol consumption. The above-mentioned physiological responses have resulted from chronic alcohol use. After acute consumption, a 15% increase in triglycerides was observed 1 h after the ingestion of a moderate dose of alcohol during a standard dinner (15). In this context, there is no investigation about the effect of acute alcohol consumption on blood triglycerides concentration in individuals subjected to exercise.

Thus, the aim of this study was to investigate if the ingestion of a moderate dose of alcohol modulates metabolic responses during treadmill running at different fixed speeds. We also investigated the effects of this alcohol intake on urine specific gravity, an indirect marker of hydration status, and on the heart rate and rating of perceived exertion (RPE), psychophysiological responses related to exercise intensity.

## Material and Methods

### Subjects

Eight healthy male volunteers (25±6 years, 75.2±8.0 kg, 174.3±5.6 cm, 17.5±4.5% body fat), who occasionally drank alcoholic beverages, participated in this study. After being fully informed of the risks associated with the experimental procedures, subjects gave their written informed consent to participate in the study, which was approved by the Research Ethics Committee of the Universidade Federal do Recôncavo da Bahia (protocol number 2.068.354/2017). This study was performed in accordance with the ethical standards of the Helsinki Declaration and in agreement with the norms established by the Brazilian National Health Council for conducting research with humans (Resolution 466/2012).

### Experimental procedures

All volunteers were initially familiarized with the procedures that were performed in the trials. At least 48 h after familiarization, the volunteers returned to the laboratory to perform anthropometric measurements and a running speed test to identify the intensity that corresponded to a lactate concentration of 4 mM. This test consisted of running on a treadmill without inclination, starting from a speed of 8 km/h and with speed increments (1 km/h) every 1 km.

Before increasing the speed (i.e., at every 1 km), the test was interrupted for 1 min to determine the blood lactate concentration by reflectance photometry using reagent test strips inserted into a portable lactometer (Accutrend Plus, Roche Diagnostic, Switzerland). This measurement was made within a maximum of 1 min after interrupting the test. Fingertip capillary blood samples were collected using a lancet to pierce the finger. If the recorded blood lactate concentration was below 4 mM, the treadmill speed was increased by 1 km/h the number of times required to reach a concentration of 4 mM. Even after identifying the speed corresponding to this concentration, there was a further 1-km run at the next speed to ensure that the recorded value would be greater than 4 mM. Although determining the exercise intensity associated with a concentration of 4 mM is used as a method to estimate the onset of blood lactate accumulation (16), we do not use this terminology in the present study, as this threshold lactate concentration disregards individual variability (17). In the 24 h prior to this test and the two experimental trials, the volunteers were instructed to avoid practicing vigorous physical exercise and to refrain from ingesting alcohol and caffeine. The volunteers were also instructed to record food and beverage intake during the 24 h prior to the first trial and to replicate this intake before the second trial.

Once the speed corresponding to 4 mM was determined, the order of the trials was balanced, whereby half of the volunteers (n=4) started the experiment with the alcohol trial and the other half (n=4) started with the non-alcohol, control trial. Each volunteer performed the two trials always at the same time of day. In one of the trials, the volunteers ingested 0.4 g ethanol/kg body weight (Smirnoff vodka), diluted in orange juice at a ratio of 1.0/3.2. This alcohol concentration was chosen because it represents half of a dose considered high that, according to El-Sayed et al. (18), corresponds to 0.8 g ethanol/kg body weight. In the other trial, they only ingested orange juice (i.e., a more concentrated juice), whereby the solution had the same calorie and volume of the solution ingested during the alcohol trial. The juice ingested in both trials was prepared using a sweetened orange juice powder (Tang).

On the day of the trials, the volunteers arrived at the laboratory in a fed state and blood was drawn from the capillaries of the fingertip directly to reagent test strips to determine the blood concentrations of lactate, triglycerides, and glucose before the solution was ingested. Measurements of triglycerides and glucose concentrations were performed using the same procedure and equipment as those described for lactate, but using reagent strips that are specific for each blood parameter analyzed. Immediately after these measurements, the volunteers ingested the solution corresponding to the experimental day and remained at rest. Twenty min after ingesting the solution, the individuals urinated in a disposable cup to determine the urine specific gravity with the help of a portable refractometer (model ITREF-200, Instrutemp, Brazil).

Thirty min after the solution intake, blood was drawn from the capillaries of the fingertip to determine the blood concentrations of lactate, triglycerides, and glucose before exercise. Next, the volunteers performed two 1-km treadmill runs in the following order: 1) speed corresponding to a blood lactate concentration of 4 mM (S4mM); and 2) speed 15% above the speed corresponding to a blood lactate concentration of 4 mM (15% above S4mM). During the exercise protocol, the heart rate and the RPE were recorded every 200 m through a heart rate monitor (model RCX5, Polar Electro, Finland) and the Borg's RPE scale (19), respectively.

At the end of the first 1-km run, a blood sample was taken from the fingertip to determine the blood lactate and triglycerides concentrations. At the end of the second 1-km run and, therefore, at the end of the experimental day, another blood sample was taken from the fingertip to determine the lactate, triglycerides, and glucose concentrations. Blood was drawn within a maximum of 1 min after finishing the run and the interval between the running protocols was 7 min of passive rest. After the last blood sample was taken, the volunteers collected another urine sample in a disposable cup to determine again their hydration level. The volunteers were not allowed to drink water while running on the treadmill or during the interval between the two running sessions.

The environmental conditions of the experimental room were measured with a thermo-hygrometer (model ITHT 2250, Instrutemp). The ambient temperature (non-alcohol: 26.5±0.3°C *vs* alcohol: 26.4±0.3°C; P=0.45) and relative humidity (non-alcohol: 47.5±2.7% *vs* alcohol: 48.3±1.7%; P=0.76) were not different between experimental trials.

### Statistical analysis

The Shapiro-Wilk and Levene tests were used to assess data normality and homoscedasticity, respectively. The concentrations of lactate, glucose, and triglycerides and the urine specific gravity were compared between experimental trials and time-points using repeated-measures two-way analyses of variance (ANOVAs) followed by Tukey's *post hoc* tests, whenever applicable. The beverage-induced (i.e., differences between post- and pre-consumption) and the exercise-induced (i.e., differences between post- and pre-exercise) changes in lactate and triglycerides concentrations were compared between experimental trials using paired Student's *t*-tests.

The parameters measured during the running protocol (i.e., heart rate and RPE) were compared between experimental trials, exercise intensities, and distance travelled using three-way ANOVA, followed by Tukey's *post hoc* tests, whenever applicable. Because the literature does not provide a non-parametric test that is analogous to a three-way ANOVA, we analyzed the RPE as if this variable had a normal distribution. All data are reported as means±SD, and the level of significance adopted was α<0.05. All analyses were performed using the SigmaPlot software (version 11.0, Systat Software Inc., USA).

The effect sizes for comparisons between pairs of means (i.e., Cohen's d_z_; within-subjects design) were calculated using the G*Power software (version 3.1.9.2, Universität Düsseldorf, Germany). The effect size values were classified as trivial (ES<0.2), small (ES=0.2-0.6), moderate (ES=0.6-1.2), or large (ES≥1.2) (20).

## Results

The concentrations of lactate, triglycerides, and glucose measured before the beverage ingestion (i.e., baseline concentrations) did not differ between the experimental trials, with all P-values being higher than 0.69.

**Figure 1:**
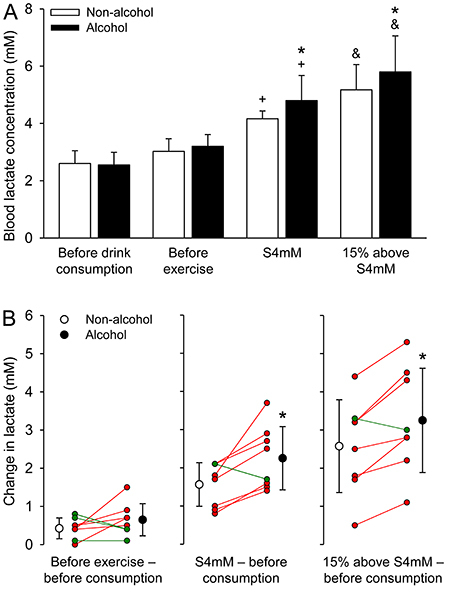
Blood lactate concentration during exercise after the intake of alcohol or a control drink. **A**, Lactate was measured at four different time-points: before drink consumption, before exercise (after drinking consumption), exercise speed corresponding to a lactate concentration of 4 mM (S4mM), and exercise speed 15% above S4mM. The two-way repeated measures ANOVA yielded the following results: trial effect (F=7.451, P=0.029); time-point effect (F=33.523, P<0.001); interaction between the two factors (F=4.060, P=0.020). Data are reported as means±SD. **B**, Changes in lactate concentration induced by the ingestion of the drinks and by the two exercise intensities relative to baseline values. The paired *t*-test yielded the following results: ingestion of the drinks (P=0.244), S4mM (P=0.018), and 15% above S4mM (P=0.009). *P<0.05 relative to the non-alcohol trial. ^+^P<0.05 relative to before drink consumption and to before exercise. ^&^P<0.05 compared to the other three time-points (n=8 volunteers in each experimental trial). Red and green lines indicate, respectively, the individuals in whom alcohol enhanced and attenuated (or did not change) the exercise-induced increase in lactate concentration.

The blood lactate concentration measured 30 min after the beverage intake was not influenced by alcohol ingestion (non-alcohol: 3.0±0.4 mM *vs* alcohol: 3.2±0.4 mM; P=0.376; d_z_=0.52) (Figure 1A). During the exercise trials, all the eight volunteers experienced increases in their blood lactate concentrations relative to concentrations recorded at resting conditions (e.g., a 62%-increase when comparing concentrations at rest and at S4mM in the control trial). In the present study, the speed corresponding to S4mM was 9.7±1.9 km/h, whereas the speed corresponding to 15% above S4mM was 11.3±2.2 km/h. Alcohol intake increased blood lactate concentration by 14.3% after exercise at the intensity corresponding to S4mM (non-alcohol: 4.2±0.3 mM *vs* alcohol: 4.8±0.9 mM; P=0.003; d_z_=0.87) and by 11.5% at the intensity corresponding to 15% above S4mM (non-alcohol: 5.2±0.9 mM *vs* alcohol: 5.8±1.3 mM; P=0.004; d_z_=0.83). These increases in lactate concentration induced by alcohol can be classified as moderate effect sizes. Remarkably, the greater exercise-induced increase in blood lactate was observed in seven of the eight volunteers after ingesting alcohol regardless of the running intensity; only one volunteer (i.e., the same subject in both exercise intensities) did not present the alcohol-mediated increase in blood lactate (Figure 1B).

**Figure 2:**
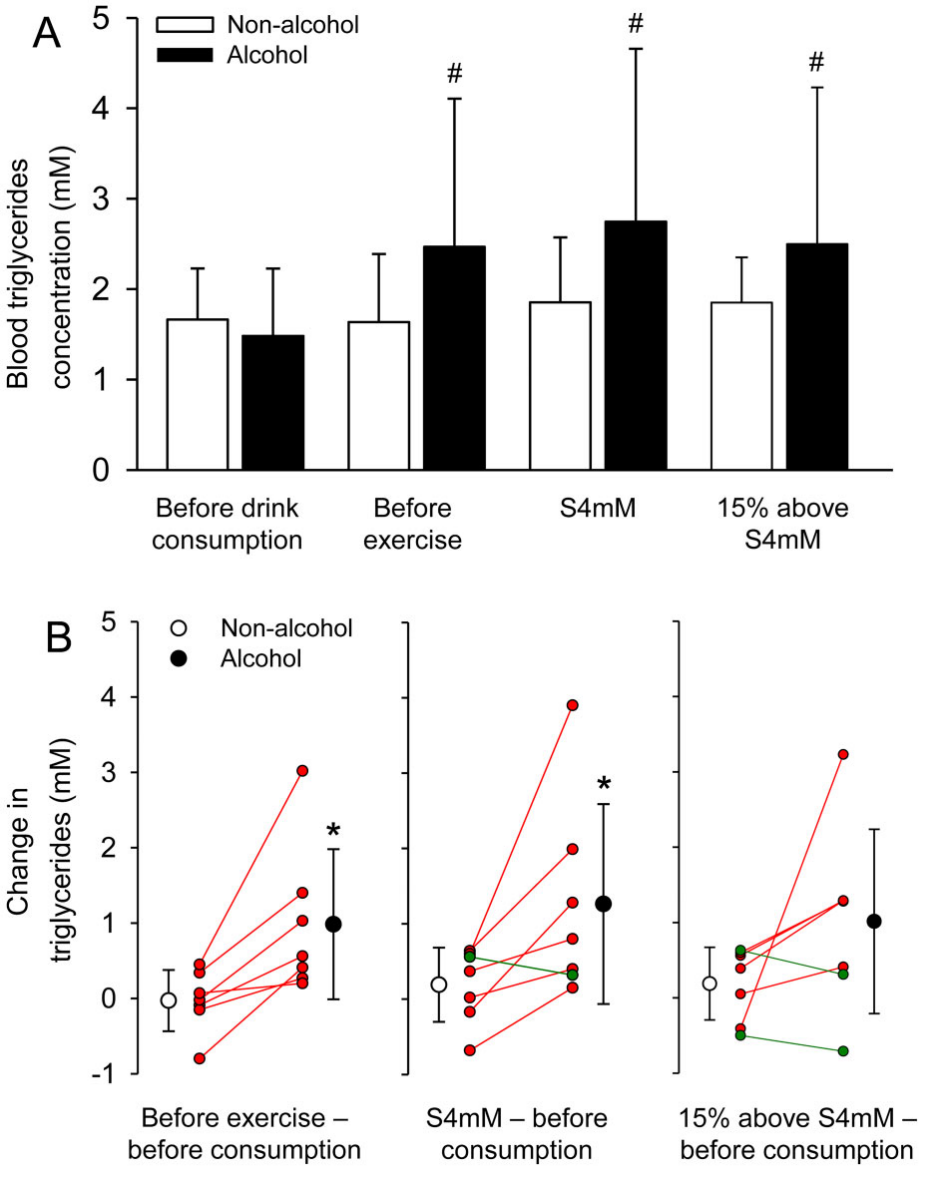
Blood triglycerides concentration during exercise after the intake of alcohol or a control drink. **A**, Triglycerides concentration was measured at four different time-points: before drink consumption, before exercise (after drinking consumption), exercise speed corresponding to a lactate concentration of 4 mM (S4mM), and exercise speed 15% above S4mM. The two-way repeated measures ANOVA yielded the following results: trial effect (F=1.894, P=0.218); time-point effect (F=4.493, P=0.016); interaction between the two factors (F=4.160, P=0.022). Data are reported as means±SD. **B**, Changes in triglycerides concentration induced by the ingestion of the drinks and by the two exercise intensities relative to baseline values. The paired *t*-test yielded the following results: ingestion of the drinks (P=0.015), S4mM (P=0.048), and 15% above S4mM (P=0.151). ^#^P<0.05 relative to before drink consumption. *P<0.05 relative to the non-alcohol trial (n=7 volunteers in each experimental trial). Red and green lines indicate, respectively, the individuals in whom alcohol augmented and reduced the exercise-induced change in triglycerides concentration.

An alcohol-mediated effect was also observed in triglycerides concentration (Figure 2A). However, although a significant interaction between trials and time-points was reported for blood triglycerides concentration (F=4.160, P=0.022), the *post hoc* test did not identify differences between the experimental trials before and after exercise. Notably, relative to baseline values, triglycerides concentration increased by 66.7% 30 min after acute alcohol intake, with higher concentrations being observed in all volunteers. An increase in triglycerides was also observed after exercise performed under the effect of alcohol (baseline: 1.5±0.7 mM *vs* S4mM: 2.7±1.9 mM; P<0.001; d_z_=0.89), a response that was not shown in the experimental trial without alcohol consumption (baseline: 1.7±0.6 mM *vs* S4mM: 1.9±0.7 mM; P=0.879; d_z_=0.41) (Figure 2A). Therefore, we decided to calculate the changes in triglycerides induced by beverage intake (Figure 2B) and then observed a greater increase caused by the alcohol compared to the control trial (non-alcohol: 0.0±0.4 mM *vs* alcohol=1.0±1.0 mM; P=0.015; d_z_=1.26). We also observed a running-induced increase after the ingestion of alcohol at the intensity of 4 mM (non-alcohol: 0.2±0.5 mM *vs* alcohol=1.3±1.33 mM; P=0.048; d_z_=0.98), but not at the intensity of 15% above 4 mM (non-alcohol: 0.2±0.5 mM *vs* alcohol=1.0±1.2 mM; P=0.151; d_z_=0.61). The latter results were classified as large (before exercise) and moderate (after exercise) magnitude effects. At the intensity corresponding to 4 mM, six of the seven volunteers presented a greater exercise-induced increase in blood triglycerides after ingesting alcohol (Figure 2B).

**Figure 3:**
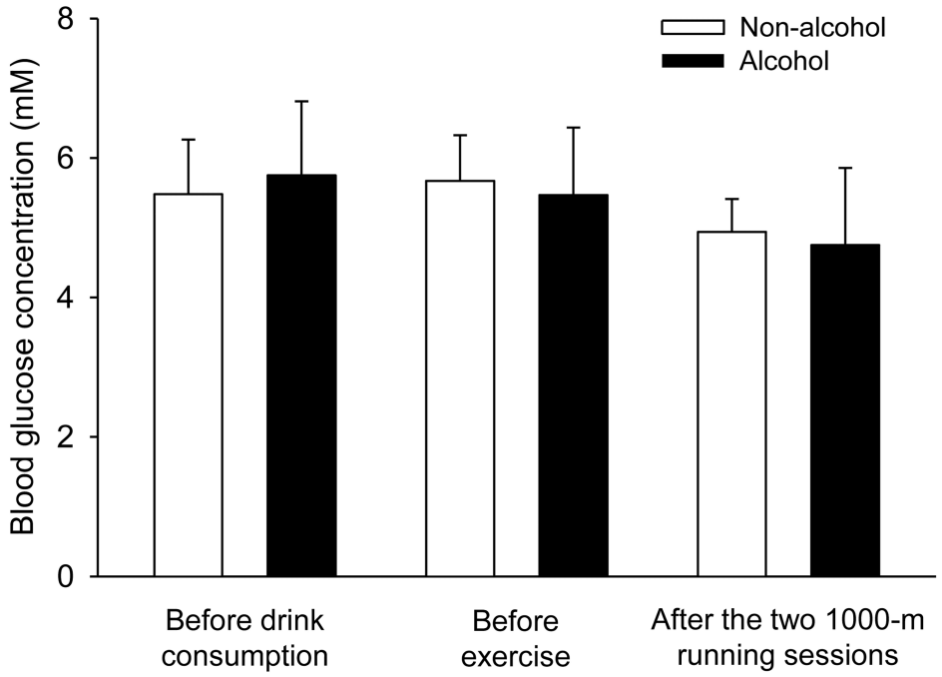
Blood glucose concentration during exercise after the intake of alcohol or a control drink. Glucose was measured at three different time-points: before drink consumption, before exercise (after drinking consumption), and at the end of the two 1000-m running sessions. The two-way repeated measures ANOVA yielded the following results: trial effect (F=0.044, P=0.840); time-point effect (F=2.818, P=0.094); interaction between the two factors (F=1.346, P=0.292). Data are reported as means±SD (n=8 volunteers in each experimental trial).

The blood glucose concentration measured 30 min after the beverage intake was not different between experimental trials (non-alcohol: 5.7±0.7 mM *vs* alcohol: 5.5±1.0 mM; P=0.466; d_z_=0.26) (Figure 3). Although glucose concentration was reduced by 17 and 11% at the end of the exercise protocol (relative to baseline values) after the consumption of alcohol and the control drink, respectively, glucose concentration at the end of the protocol was not influenced by alcohol ingestion (non-alcohol: 4.9±0.5 mM *vs* alcohol: 4.8±1.1 mM; trial effect, P=0.587; d_z_=0.11) (Figure 3).

**Figure 4:**
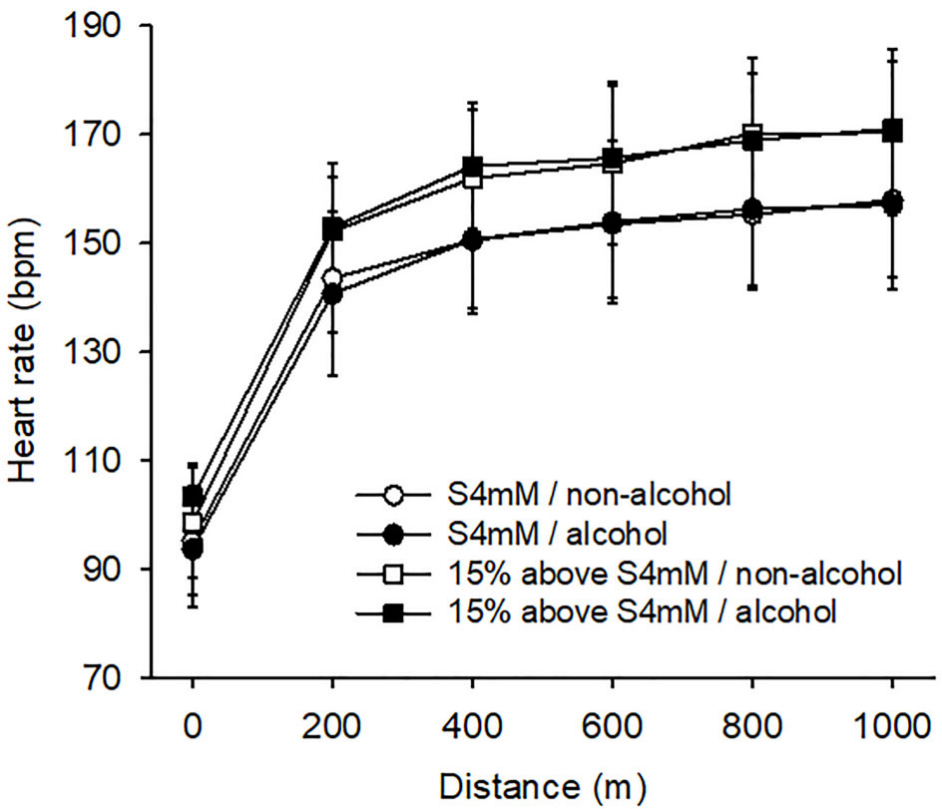
Heart rate during exercise after the intake of alcohol or a control drink. Heart rate was measured at 200-m intervals during exercise performed at the speed corresponding to a lactate concentration of 4 mM (S4mM) and at the speed 15% above S4mM. The three-way repeated measures ANOVA yielded the following results: distance travelled effect (F=125.671; P<0.001); intensity effect (F=37.365, P<0.001); trial effect (F=0.041, P=0.839); no interactions were observed between pairs of factors or between the three factors (P>0.05). Data are reported as means±SD (n=8 volunteers in each experimental trial).

As expected, the heart rate and RPE increased gradually with the distance travelled. In addition, running at 15% above S4mM represented greater cardiac strain and perceived exertion compared to running at S4mM; however, alcohol intake did not influence heart rate (Figure 4) and RPE (Table 1) responses at either speed.

**Table 1:** Rating of perceived exertion (RPE) measured at 200-m intervals during exercise performed at S4mM and at 15% above S4mM after the intake of alcohol or a control drink.

Intensity/trial	Distance				
	200 m	400 m	600 m	800 m	1,000 m
S4mM					
Non-alcohol	8±2	9±2	10±2	10±2	11±2
Alcohol	8±1	9±2	9±2	10±3	10±3
15% above S4mM					
Non-alcohol	9±2	10±2	11±3	12±3	13±3
Alcohol	9±2	10±3	11±2	12±3	13±3

RPE was compared between trials, exercise intensities, and distance travelled using a three-way ANOVA, which yielded the following results: distance travelled effect (F=8.169, P<0.001); intensity effect (F=16.887, P<0.001); trial effect (F=0.477, P=0.491); no interactions were observed between pairs of factors or between the three factors (P>0.05) (n=8 volunteers in each experimental trial). Data are reported as means±SD.

Compared to the pre-exercise values, the volunteers finished the exercise sessions with lower urine specific gravity in the alcohol trial (before: 1.026±0.006 *vs* after exercise: 1.011±0.005; P<0.001; d_z_=12.67), but with similar values in the orange juice trial (before exercise: 1.030±0.007 *vs* after exercise: 1.029±0.008; P=0.582; d_z_=0.46). The urine specific gravity was not different between groups before exercise (P=0.218; d_z_=0.62), although the volunteers finished the exercise sessions with lower gravity in the alcohol trial (non-alcohol: 1.029±0.008 *vs* alcohol: 1.011±0.005; P<0.001; d_z_=1.58).

## Discussion

This is the first study to demonstrate that pre-exercise ingestion of a moderate dose of alcohol increased lactate concentration during a fixed-intensity exercise, relative to the ingestion of a non-alcoholic solution. Interestingly, the acute alcohol intake increased triglycerides concentration already under resting conditions (i.e., 30 min after drinking vodka) and this response was sustained during the subsequent exercise session. Alcohol intake also decreased urine specific gravity but did not affect blood glucose concentration, heart rate, and RPE.

The moderate dose of alcohol administered in the present study was sufficient to increase the blood lactate concentration by 11–14% at the two running speeds investigated. This information is very relevant for people involved in sports (e.g., coaches and strength and conditioning practitioners), as athletes often consume alcohol because of psychological, cultural, and social reasons and even to relieve the tensions generated by training and competitions (21). Indeed, athletes seem to consume much higher alcohol amounts (22,23) than the amount used herein.

There is evidence that acute alcohol consumption impairs muscular endurance, power, strength, and speed, as well as cardiovascular endurance (2). Indeed, the findings by McNaughton and Preece (5), Kendrick et al. (6), and Lecoultre and Schutz (7) strongly suggest that low to moderate doses of alcohol decrease endurance performance. Our findings highlight that changes in muscular metabolism are likely one of the reasons that could explain the decreased performance caused by alcohol. A higher blood lactate concentration is suggestive of greater energy transformation through anaerobic pathways during physical exercise. This greater anaerobic contribution would reduce the running intensity associated with lactate threshold and/or the onset of blood lactate accumulation. In this sense, several studies have reported the ability of these lactate-related thresholds in predicting endurance performance (24–26). However, the latter hypothesis is contrasted by biochemical evidence showing that acidosis is caused by reactions other than lactate production; e.g., every time ATP is broken down to ADP and Pi, a proton is released (27). In addition, evidence indicates that lactate production retards, not causes, acidosis (27).

The increased blood lactate concentration caused by alcohol is likely a consequence of greater synthesis in skeletal muscles and/or reduced clearance of this metabolite from the bloodstream. Lactate clearance occurs through oxidation in skeletal muscles and also through oxidation in the cardiac muscle (28). In the heart, oxidative energy production from lactate is higher than from glucose both at rest and during moderate intensity exercise (28). In addition, the liver and kidney are also involved in the uptake of lactate from circulation; for example, lactate taken up can account for more than 50% of renal gluconeogenesis (28). Alcohol intake has been implicated in the inhibition of the Cori cycle (9), which may lead to a reduced clearance of this metabolite from circulation. However, the evidence that alcohol did not reduce glucose concentration before and after the exercise protocol (Figure 3) suggests that liver (or kidney) gluconeogenesis was not inhibited.

It is likely that the higher blood lactate concentration may be explained by a higher production. In fact, alcohol oxidation increases the NADH-to-NAD ratio (29), which in turn increases the lactate-to-pyruvate ratio and causes hyperlactatemia (10). Moreover, the reduced NAD levels impair the conversion of lactate to pyruvate (9) and decelerate the citric acid cycle, thereby decelerating the aerobic metabolism (10). If a slower aerobic metabolism indeed occurred, there may have been an increase in the participation of the glycolytic pathway in transforming the energy required for muscle contractions, which results in greater lactate production. To test this hypothesis, future studies should investigate whether an acute alcohol intake modulates the oxygen consumption and running economy during similar exercise protocols as those used in this study.

It has been suggested that the hypoglycemic effect of alcohol is among the factors that may decrease endurance performance (6). In the present study, the exercise-induced reduction in glucose concentration in the alcohol trial was lower than that shown by Kendrick et al. (6) (17 *vs* 24%). The shorter exercise duration and the lower exercise intensity protocol used in the present study may explain the differences between the two studies. Despite the differences in the severity of hypoglycemia, the reduction induced by alcohol intake was not different relative to the non-alcohol trial in ours and in Kendrick's study.

No differences in cardiovascular strain and in the sense of effort were observed after alcohol intake, as evidenced by the lack of differences in the heart rate (Figure 4) and RPE (Table 1) between trials. Our results regarding cardiac strain are in accordance with the ACSM Position Stand (2), which reported that acute alcohol use has no detrimental effect on the heart rate during exercise. With respect to the perceived exertion, evidence indicates that low doses of alcohol induce dopamine release in part of the nucleus accumbens, a brain region involved in motivation and reinforcement (30). Particularly, oral alcohol administration influences dopamine release both through its gustatory properties and through its direct actions on the brain (30). Previous investigations reported that athletes who ingested an inhibitor of dopamine reuptake presented greater exercise-induced increases in core body temperature, without concomitant changes in RPE or perception of thermal stress during a time trial (31). Thus, we suggest that alcohol caused the release of dopamine, which acted centrally to maintain a given RPE, despite the observed physiological changes outside the central nervous system (i.e., higher lactate concentration). An alternative hypothesis to explain the lack of alcohol effect on RPE is the idea that afferent feedback from skeletal muscles and other peripheral tissues does not contribute significantly to perceived exertion, which has been considered the conscious awareness of the central motor commands to the locomotor and respiratory muscles (32).

Our data showed an increase in blood triglycerides concentration before and during exercise in the alcohol trial (Figure 2), whereas this concentration remained unaltered during the control trial. More specifically, the moderate alcohol consumption combined or not with exercise increased blood triglycerides. These results agree with findings reported after the ingestion of a moderate dose of alcohol during a standard dinner (15), but they disagree with findings observed after chronic consumption or with the expected effect of a single exercise bout. There is growing evidence that low to moderate alcohol consumption habits decrease plasma triglycerides at resting conditions (33–35). Similarly, an aerobic exercise bout has been shown to reduce serum triglycerides (36). We speculate that physiological responses, such as increased VLDL secretion, impaired lipolysis, and increased delivery of free fatty acids from adipose tissue to the liver (37) may help to explain this higher blood triglycerides concentration. Although we did not measure blood or breath alcohol concentration, the increase in blood triglycerides caused by alcohol under resting conditions indicated that the treatment itself was effective in changing metabolism.

Alcohol intake also reduced the urine specific gravity, suggesting that our volunteers experienced a marked increase in urine flow, which could be partially explained by lower secretion of anti-diuretic hormone (i.e., arginine vasopressin) induced by alcohol (38). Thus, one could argue that the higher blood lactate concentration is an artificial result caused by reduced plasma volume; nevertheless, this reasoning seems to be unlikely as no changes occurred in hematocrit and serum hemoglobin in the two hours after drinking whiskey (39). Additionally, urine values can provide misleading information regarding hydration status if obtained during rehydration periods (40); this may also be the case for a person that ingests alcohol.

According to Sawka et al. (40), urine specific gravity values equal to or lower than 1.020 are indicative of euhydration. Therefore, the values of urine gravity measured at baseline conditions suggest that some volunteers may have started the exercise in a dehydrated state, even though they have ingested 428±45 mL (means±SD) of solution 30 min before exercise initiation. This is a limitation of the present study. However, because the urine specific gravity was not different between experimental trials before exercise initiation and because the duration of the exercise protocol was very short, we believe that the fact that some volunteers were likely dehydrated when they started running does not compromise the reliability of our findings. Moreover, aerobic performance, which is impaired by dehydration (40), was not determined in the present study.

We conclude that an acute ingestion of a moderate dose of alcohol modulated the changes in lactate metabolism induced by a fixed-intensity treadmill running. In addition, alcohol intake increased triglycerides concentration already under resting conditions and this increase was sustained during the subsequent exercise session. Of note, glycemia was not modified by either alcohol intake, exercise, or their combination. Alongside these metabolic changes, an acute alcohol intake decreased urine specific gravity, but did not affect cardiac strain and perceived exertion.
